# Dr. Sugrue's Case of Gastrodynia

**Published:** 1800-09

**Authors:** Charles Sugrue

**Affiliations:** Corke


### *To the Editors of the Medical and Phyfical Journal.*

GENTLEMEN,

THE appearances after death obferved in the following cafe, are, I think, worthy of being preferved in your valuable publication, particularly as nothing fimilar is to be found in the writings of De Haen, Morgagni, or Doctor Baillie. I fhall barely enumerate fuch particulars of the cafe, as may ferve to fhew how eafily the caufe and feat of the difeafe might be mifconceived.

Richard Montjoy, aged 8 years, a thin, fprightly boy, of a fair complexion, with blue eyes, delicate fkin, and fair hair, was attacked, in the month of June of the year 1796, with a fevere pain under the ftomach. As moft of the difeafes of children, in this as well as in other countries, are afcribed to worms, fome *worm-powders* were procured from an apothecary in this city, which operated ftrongly. No mitigation of the pain pain having however enfued, I was fent for, and found the boy apparently in great agony; he complained of excruciating and unremitting pain about the lower part of the ftomach, fhooting acrofs the abdomen; his countenance was pale and languid, and his pulfe feeble. The pain was neither increafed nor diminifhed on preffure; and as the bowels were free, there was no reafon to fufpect an inflammation of any part of the inteftinal canal. Different means were tried, with a view of giving temporary eafe, but no material relief was experienced from any, except from laudanum in pretty large dofes; he continued feveral weeks in this ftate, taking very little nourifhment, and complaining of conftant pain.

On being queftioned relative to the caufe of his complaint, he could affign none, but recollected having received a fevere blow of a rattan acrofs the loins from his fchool-mafter. About the commencement of the following winter, after he had renounced the aid of medicine as fruitlefs, his health began fuddenly to mend, and he experienced temporary ceffation of pain. Thofe fymptoms became gradually more favourable, until he at length entirely recovered.

From the winter of 1796 until the month of Auguft 1799, he continued in perfect health. About the end of this month the pain about the ftomach returned, without any apparent caufe, and precifely in the fame fituation as before; it was flight at firft, but increafed daily: His appetite was at firft very bad, but afterwards became voracious; on taking food, however, the pain was much aggravated, but he felt relief from bending his body forwards. He was often attacked with diarrhœa, of the appearance of blood and pus; at other times the bowels were conftipated. He was bliftered, and took a variety of medicines, without any benefit.

Though during the three or four laft months of his illnefs he took a confiderable quantity of nourifhment, he vifibly became more emaciated and feeble. His pulfe was, during this time, about 120. His countenance refembled that of a perfon in the laft ftage of a pulmonary confumption; it was pale, and the hectic flufh was diftinctly marked on each cheek: he had conftant, profufe, and univerfal perfpiration; the abfence of cough, however, during his whole illnefs, together with the feat of the pain, left me no room to doubt that the lungs were not the feat of the difeafe.

About three months after the firft attack, the pain became gradually milder, and his appetite returned; he was, however, ftill much emaciated, and never totally free from pain. He was bliftered, and took mercury until the mouth was affected, without any advantage.

He ſpoke of the torments he endured rather in a plaintive than a querulous tone; he bore them, indeed, with a greater degree of reſignation than could be expected at his age. He ſeemed ſtrongly impreſſed with the idea that he would never recover, and frequently told his parents that he knew full well they could form no idea of the degree of his ſufferings.

About the middle of laſt May he expired; a few hours before his death he had eaten ſome veal for dinner.

His parents were anxious that the body ſhould be opened, in order that, if poſſible, the cauſe of his diſeaſe might be diſcovered. I conſequently called on Dr. Bullen, Dr. F. Walſh, and Dr. John Milner Barry, of this city, for that purpoſe.

After making a conical inciſion, and expoſing to view the viſcera of the abdomen, their appearance was quite natural; no omentum could however be obſerved, as it probably had been waſted away during his tedious illneſs: The liver appeared rather prominent, but quite ſound; the ſpleen and ſtomach exhibited no marks of diſeaſe; the inteſtines appeared natural, and no worms were found in their cavity; the meſenteric glands were very little enlarged.

On raiſing up the liver, immediately under it appeared a large, oblong tumour, which at the firſt view appeared to be a conſiderable enlargement of that portion of the colon, which after aſcending above the anterior part of the right kidney, paſſes under the liver and gall-bladder. On tracing, however, the inteſtines from the pylorus to the rectum, the canal was found quite natural, both with reſpect to diameter and appearance. On minute inveſtigation, we found this tumour attached to the right kidney, and extending from it to the diaphragm, to which it adhered by cellular ſubſtance. Having carefully diſſected it out of the abdomen, we ſeparated the kidney, which did not exhibit the leaſt appearance of diſeaſe. The tumour was of an oblong ſhape, about ſix inches long, and from two and a half to three in its largeſt diameter; it was of the ſame colour with the inteſtines externally; it was of a firm conſiſtence, and on cutting into it, we found it compoſed of a yellowiſh-coloured ſubſtance, with a ſhade of brown, not unlike the remains of beef-ſuet, after it has been melted and the tallow ſqueezed out of it. After being ſeparated from the kidney, it weighed 2¼ ℔. We had now no heſitation in pronouncing this tumour to be a conſiderable enlargement of the *capſula renalis.*

On raiſing up the ſtomach and ſpleen, a ſimilar tumour was perceived on the left ſide, which on examination was found to be the *capſula renalis* of that ſide alſo conſiderably enlarged; it reſembled in appearance that of the right ſide, but was much more more firmly connected with the diaphragm. On one of its surfaces the pancreas was found firmly attached to it, and was thus removed from its transverse situation in the abdomen, by which it is parallel to the stomach, to form an acute angle with that organ. The pancreas was not in the least enlarged, nor scirrhous, as there was some reason to expect. As so firm an adhesion between it and the tumour must have been the consequence of inflammation, to this may be referred the acute pain felt at the commencement of the disease. The capsula renalis of the left side, together with the kidney, which was of the natural size, weighed 3 ¼ ℔.

On opening the thorax, the lungs were found quite sound, and no marks of scrophula could be discovered.

CHARLES SUGRUE, M. D.

*Corke, June* 26, 1800.

